# Indoor window ventilation and anxiety symptoms among older adults with disabilities in China: A cross-sectional study

**DOI:** 10.1097/MD.0000000000047720

**Published:** 2026-02-28

**Authors:** Qiuhua Zhang, Qi Zheng, Zishuo Huang, Tingke Xu

**Affiliations:** a Department of Health Information, Zhejiang University School of Medicine Sir Run Run Shaw Hospital, Hangzhou, Zhejiang, China; b School of Public Health, Hangzhou Medical College, Hangzhou, Zhejiang, China; c School of Medical Humanities and Management, Wenzhou Medical University, Wenzhou, Zhejiang, China.

**Keywords:** anxiety symptoms, Chinese Longitudinal Healthy Longevity Survey, Chinese older adults with disabilities, indoor window ventilation

## Abstract

Although prior research indicated that indoor ventilation might have supported mental health in older adults, evidence remained scarce for those with disabilities, who represented a particularly vulnerable population. This study investigated the association between indoor window ventilation frequency and anxiety symptoms among older adults with disabilities in China using nationally representative data. A cross-sectional analysis was conducted using the Chinese Longitudinal Healthy Longevity Survey. Anxiety symptoms were assessed using a validated scale, and indoor window ventilation frequency was examined across multiple seasons. Multivariable linear regression models were used to evaluate associations. Frequent indoor window ventilation was significantly associated with lower anxiety symptoms. Compared with low frequency ventilation, medium (*B* = −0.651, 95% confidence interval = −1.119, −0.183) and high (*B* = −0.793, 95% confidence interval = −1.243, −0.342) frequency ventilation showed stronger protective effects. Seasonal ventilation patterns also demonstrated consistent negative associations with anxiety symptoms. This study added scientific value by identifying a low-cost, environmental strategy to alleviate anxiety in disabled older adults. Our findings called for greater policy attention to indoor ventilation practices, including public awareness campaigns and improvements to living environments to promote mental health in aging populations.

## 1. Introduction

Mental health is increasingly recognized as a cornerstone of global public health.^[1]^ Among various mental disorders, anxiety stands out due to its high prevalence, complex symptomatology, and substantial societal burden – including an estimated annual cost of USD 1 trillion and its strong association with suicide and overall mortality.^[2]^ Anxiety disorders commonly co-occur with other physical and psychological conditions, manifesting through heightened stress responses and a range of somatic symptoms.^[3–5]^ These characteristics underscore the importance of investigating environmental and behavioral factors that may mitigate anxiety symptoms.

As global populations age, late-life anxiety has emerged as an urgent public health issue, especially among older adults with disabilities.^[6]^ This demographic faces compounding vulnerabilities – reduced mobility, multiple chronic illnesses, medication dependence, and social isolation – that exacerbate both physical and psychological distress.^[7]^ China, home to the world’s largest aging population, is projected to host over 120 million disabled older adults by 2050.^[8]^ The magnitude of this demographic shift necessitates scalable, preventive strategies to support mental well-being in this high-risk group.

Indoor window ventilation serves not only as a physical means of removing indoor air pollutants but also as a psychological bridge connecting occupants to the natural environment. Accumulating evidence suggests that visual engagement with outdoor landscapes – such as greenery, daylight, and open skies – has measurable benefits for mental health, especially in older adults.^[9]^ Viewing natural scenery has been associated with enhanced attention restoration and reduced symptoms of anxiety, which are frequently observed in aging populations.^[10,11]^

Simultaneously, poor indoor air quality – marked by elevated levels of fine particulate matter, ozone, and other pollutants – has been linked to increased psychological distress, including heightened anxiety among elderly individuals.^[12]^ Older adults with disabilities are particularly susceptible, as their limited mobility leads to longer periods spent indoors, compounding their exposure to suboptimal indoor environments.^[13]^ Window ventilation, as a habitual behavior, may help mitigate these risks by improving air exchange and reducing pollutant concentrations. Some studies have also associated ventilation practices with improved cognitive outcomes in older populations.^[14]^

However, despite growing recognition of the environmental determinants of mental health, little is known about how the frequency of indoor window ventilation specifically influences anxiety symptoms among disabled older adults in China – a population both demographically significant and understudied in mental health research. Existing literature has primarily focused on general older populations or objective air quality metrics, without examining the behavioral dimension of ventilation in daily residential life.

To address this gap, our study investigates the association between indoor window ventilation frequency and anxiety symptoms in disabled older Chinese adults using data from the nationally representative 2018 Chinese Longitudinal Healthy Longevity Survey (CLHLS). This approach allows for a nuanced understanding of how everyday environmental practices may support mental well-being in a highly vulnerable segment of the population.

## 2. Methods

### 2.1. Data sources and study sample

Our study employed a cross-sectional design, utilizing data from the 2018 CLHLS, developed by the Center for Healthy Aging and Development Research at Peking University. Since its inception in 1998, CLHLS has been conducted across 23 provinces, municipalities, and autonomous regions in China, encompassing extensive microdata on various dimensions of aging. These include physical and mental health, cognitive functioning, social participation, behaviors, dietary and nutritional habits, lifestyle factors, socioeconomic status, family structure, intergenerational relationships, elderly care needs and costs, and the health status of deceased individuals aged 65 and older prior to their passing. CLHLS data provide valuable insights into the multifaceted challenges faced by older adults during the aging process. It also evaluated the effectiveness of different social policy interventions aimed at enhancing the quality of life of the elderly. Thus, the CLHLS serves as a critical theoretical and empirical foundation for addressing aging-related challenges in China.

In this study, respondents were excluded if they had no limitations on basic activities of daily living (BADL), dearth of data in relation to the 7-item Generalized Anxiety Disorder Scale (GAD-7), indoor ventilation frequency, and other main variables. A total of 15,874 individuals participated in the 2018 CLHLS. Crucially, 11,894 respondents had no limitations of BADL, 527 respondents lacked the data of GAD-7, 172 respondents lacked data on indoor ventilation frequency, and 613 lacked the main variables, and all of them were excluded from this study. The final sample of 2352 older adults with disabilities was included in the study.

### 2.2. Measurements

#### 2.2.1. Identification of older adults with disabilities

Functional disabilities in older adults are commonly assessed using 2 measures: BADL and instrumental activities of daily living (IADL). BADL, which includes essential self-care tasks, such as bathing, dressing, eating, and toileting, serves as the most fundamental indicator of self-sufficiency compared with IADL.^[15]^ Notably, older adults with BADL typically have impaired IADL. Furthermore, research indicates that older adults with BADL disabilities are more prone to experiencing anxiety symptoms than those with IADL disabilities only. Therefore, this study utilized BADL as the defining criterion for identifying older adults with disabilities.

The Katz BADL index in the CLHLS encompasses 6 key items: bathing, dressing, toileting, indoor activities, urination and defecation, and eating.^[16]^ Older adults were classified as disabled if they had one or more limitations in their BADL. For scoring, a value of 1 indicated partial or complete inability to perform self-care tasks, whereas 0 represented full independence without requiring assistance. The total BADL score ranged from 0 to 6, with 1 to 2 signifying mild disability, 3 to 4 indicating moderate disability, and 5 to 6 reflecting severe disability.

#### 2.2.2. Evaluation of anxiety symptoms

Anxiety symptoms were measured using the GAD-7 consisting of 7 negative items. Respondents were asked how often they had experienced anxiety symptoms in the past 2 weeks. Responses of “none,” “a few days,” “over half the time,” and “almost daily” were coded 0 to 3, respectively. Anxiety symptom scores ranged from 0 to 21, with lower scores indicating lower levels of anxiety symptoms.^[17]^

#### 2.2.3. Evaluation of indoor window ventilation frequency

Respondents were asked how often they had opened windows per week in each season for the past 12 months. Each of the responses of “no times per week,” “1–5 times per week,” and “over 5 times per week” were coded 0 to 2, respectively. Total scores ranged from 0 to 8, with 0 to 3 indicating low ventilation frequency, 4 to 5 medium ventilation frequency, and 6 to 8 high ventilation frequency.^[14]^

#### 2.2.4. Covariates

In this study, we used various sociodemographic, health status, lifestyle, and environmental characteristics as covariates. Sociodemographic characteristics included sex (male, female), age (<80 years old, ≥80 years old), marital status (married, single), education (0 years: illiterate, >0 years: literate), residence (urban or rural), and self-reported income level (high or low); health status included the levels of disabilities (mildly disabled, moderately disabled, severely disabled) and self-reported health (very good, good, fair, poor, very poor); lifestyle factors included smoking (yes, no), drinking (yes, no), and exercising (yes, no).

### 2.3. Statistical analysis

Descriptive statistical methods are presented in terms of frequency, percentage, and mean ± SD. We then applied an analysis of variance to compare anxiety symptom scores among the basic characteristics of Chinese older adults with disabilities and situations of seasonal and overall indoor frequency. Multiple linear regressions were used to detect an association between indoor window ventilation frequency and anxiety symptoms in older adults with disabilities. In the subgroup analysis, correlations between seasonal indoor window ventilation frequency and anxiety symptoms were measured using multiple linear regressions after adjusting for covariates.

## 3. Results

### 3.1. Descriptive analysis

A total of 2352 disabled older adults with disabilities participated in this study. The overall mean score for anxiety symptoms was 1.83 (SD = 3.35). The findings indicated that anxiety symptoms were more prevalent among specific subgroups, including individuals aged <80 years, those who were married, those residing in rural areas, individuals with low income, those reporting poor or very poor health status, participants with relatively high levels of disability, those who did not engage in regular exercise, and individuals with lower frequencies of indoor ventilation. The detailed baseline characteristics are presented in Table 1.

**Table 1:** Basic characteristics of Chinese older adults with disabilities.

Variables	N (%)	Anxiety symptoms		
		Mean ± SD	*F*	*P*-value
Total	2352	1.83 ± 3.35		
Gender			1.365	.243
Male	830 (35.29)	1.73 ± 3.19		
Female	1522 (64.71)	1.90 ± 3.44		
Age			15.444	<.001
<80 yr	182 (7.74)	2.77 ± 4.05		
≥80 yr	2170 (92.26)	1.76 ± 3.27		
Education			1.670	.196
Illiterate	1520 (64.63)	1.90 ± 3.43		
Literate	832 (35.37)	1.71 ± 3.20		
Marital status			4.666	.031
Married	440 (18.71)	2.15 ± 3.35		
Single	1912 (81.29)	1.76 ± 3.35		
Residence			16.326	<.001
Urban	707 (30.06)	1.41 ± 2.74		
Rural	1645 (69.94)	2.02 ± 3.57		
Income			70.567	<.001
High	2014 (85.63)	1.60 ± 3.00		
Low	338 (14.37)	3.23 ± 4.71		
SRH			224.088	<.001
Very good, good, fair	1802 (76.62)	1.29 ± 2.58		
Poor, very poor	550 (23.38)	3.62 ± 4.69		
Levels of disabilities			17.545	<.001
Mildly disabled	1307 (55.57)	1.50 ± 2.87		
Moderately disabled	518 (22.02)	2.02 ± 3.61		
Severely disabled	527 (22.41)	2.49 ± 4.03		
Smoking			0.480	.489
Yes	215 (9.14)	1.68 ± 3.10		
No	2137 (90.86)	1.85 ± 3.38		
Drinking			0.037	.848
Yes	204 (8.67)	1.88 ± 3.41		
No	2148 (91.33)	1.83 ± 3.35		
Regularly exercising			5.744	.017
Yes	352 (14.94)	1.44 ± 2.90		
No	2000 (85.03)	1.90 ± 3.42		
Overall ventilation frequency			14.474	<.001
Low	233 (9.91)	2.86 ± 4.32		
Intermediate	744 (31.63)	1.93 ± 3.17		
High	1375 (58.46)	1.61 ± 3.22		

SD = Standard Deviation, SRH = self-reported health.

### 3.2. Regression analysis

Table 2 shows the findings of multiple linear regression analyses. After adjusting for covariates, the results revealed that medium indoor ventilation frequency (*B* = −0.651, 95% confidence interval [CI] = −1.119, −0.183) and high indoor ventilation frequency (*B* = −0.793, 95% CI = −1.243, −0.342) were significantly associated with reduced anxiety symptoms among older adults with disabilities in China compared with low ventilation frequency. Additionally, factors such as residence, income, self-reported health, and levels of disability were identified as significant predictors of anxiety symptoms.

**Table 2:** Multiple linear regression: factors affecting anxiety symptoms among Chinese older adults with disabilities.

Variable	*B*	95% CI	*P*-value
Gender (ref. = male)	0.178	(−0.136, 0.492)	.266
Age (ref. =<80 yr)	−0.462	(−1.010, 0.086)	.098
Education level (ref. =illiterate)	0.008	(−0.315, 0.330)	.963
Marital status (ref. =married)	−0.012	(−0.398, 0.373)	.950
Residence (ref. =rural)	0.333	(0.027, 0.639)	.033
Income (ref. =high)	1.049	(0.674, 1.423)	.000
Self-rated health (ref. =very good, good, fair)	2.002	(1.685, 2.318)	.000
Levels of disabilities (ref. =mildly disabled)			
Moderately disabled	0.339	(0.013, 0.666)	.042
Severely disabled	0.526	(0.191, 0.860)	.002
Smoking (ref. =yes)	0.154	(−0.313, 0.620)	.519
Drinking (ref. =yes)	−0.316	(−0.785, 0.154)	.188
Exercising (ref. =yes)	0.027	(−0.346, 0.401)	.885
Indoor ventilation frequency (ref. =low frequency)			
Medium frequency	−0.651	(−1.119, −0.183)	.006
High frequency	−0.793	(−1.243, −0.342)	.001

CI = confidence interval.

### 3.3. Subgroup analysis

Our findings revealed a negative association between indoor ventilation frequency and anxiety symptoms in all seasons. Specifically, a medium ventilation frequency significantly reduced anxiety symptoms during spring (*B* = −0.948, 95% CI = −1.586, −0.310), summer (*B* = −1.712, 95% CI = −2.548, −0.876), fall (*B* = −1.282, 95% CI = −1.950, −0.613), and winter (*B* = −0.423, 95% CI = −0.765, −0.080). Similarly, high ventilation frequency also demonstrated a stronger alleviating effect on anxiety symptoms in spring (*B* = −1.141, 95% CI = −1.777, −0.506), summer (B = −1.739, 95% CI = −2.547, −0.931), fall (B = −1.509, 95% CI = −2.173, −0.844), and winter (B = −0.407, 95% CI = −0.756, −0.059). These subgroup analyses align with the main results of our study, providing consistent evidence of the beneficial impact of indoor ventilation frequency in reducing anxiety symptoms. Further details are provided in Table 3.

**Table 3:** Subgroup analysis: the association between seasonal indoor frequency and anxiety symptoms.

Variable	B	95% CI	*P*-value
Spring ventilation frequency (ref. =0 time/wk)			
1–5 times/wk	−0.948	(−1.586, −0.310)	.004
>5 times/wk	−1.141	(−1.777, −0.506)	.000
Summer ventilation frequency (ref. =0 time/wk)			
1–5 times/wk	−1.712	(−2.548, −0.876)	.000
>5 times/wk	−1.739	(−2.547, −0.931)	.000
Fall ventilation frequency (ref. =0 time/wk)			
1–5 times/wk	−1.282	(−1.950, −0.613)	.000
>5 times/wk	−1.509	(−2.173, −0.844)	.000
Winter ventilation frequency (ref. =0 time/wk)			
1–5 times/wk	−0.423	(−0.765, −0.080)	.016
>5 times/wk	−0.407	(−0.756, −0.059)	.022

CI = confidence interval.

## 4. Discussion

This cross-sectional study conducted an independent assessment of the associations between overall and seasonal indoor window ventilation frequencies and anxiety symptoms among older adults with disabilities in China. Our findings demonstrate that a higher overall indoor ventilation frequency was significantly associated with a lower likelihood of anxiety symptoms in this population. Furthermore, when stratified by season, the results are consistent and reliable. To the best of our knowledge, this study is the first to investigate the relationship between indoor window ventilation frequency and anxiety symptoms among older adults with disabilities in China.

The findings of this study revealed a significant inverse relationship between indoor window ventilation frequency and anxiety symptoms among older adults with disabilities in China, even after adjusting for various covariates. Specifically, a higher frequency of window ventilation was linked to a greater likelihood of anxiety reduction. Households where disabled older adults experience frequent window ventilation are consistently exposed to sustained sensory stimuli from the outdoor environment. One possible explanation is that visual engagement with nature may act as a protective factor against anxiety in this vulnerable population.

Observing natural scenery plays a vital role in restoring attention, reducing stress, and enhancing mental health and well-being in older adults with disabilities. Recent studies have shown that visual exposure to outdoor natural environments through windows significantly improves the concentration of older adults with disabilities, thereby alleviating anxiety symptoms.^[11,18]^ Previous research has also highlighted the positive impact of viewing natural elements, such as trees, meadows, and fields on stress recovery, demonstrating a noticeable reduction in anxiety symptoms.^[9]^ Additionally, a recent study has emphasized the mental health benefits of window ventilation.^[19]^ It was found that individuals reported more frequent positive emotions (e.g., happiness and satisfaction) and fewer negative emotions (e.g., sadness and lethargy) in the presence of a window than in their absence, effectively mitigating anxiety. For disabled older adults with mobility limitations who are unable to engage regularly in outdoor environments, opening windows to view nature can serve as a simple yet effective strategy to improve attention, reduce stress, and lower anxiety levels.

Another plausible explanation is that frequent window opening for ventilation helps expel indoor humidity and pollutants, creating a healthier living environment. Improved ventilation increases indoor air infiltration rates and reduces the incidence of sick building syndrome symptoms.^[20,21]^ Proper indoor ventilation is crucial for preventing the buildup of harmful environmental hazards, such as pollutants emitted by building materials, by-products of fuel combustion, and radon gases. In addition, it lowers the risks associated with allergies, asthma, and vectors that transmit infectious viruses. This is particularly significant for disabled older adults, who are often confined to bed and experience age-related physical and mental decline, and this holds particular significance.^[22]^ Studies have indicated that approximately 80% of elderly individuals with disabilities in China suffer from one or more chronic diseases.^[23]^ Window opening for ventilation is an energy-efficient and eco-friendly housing practice that helps optimize indoor air humidity and quality, reducing the risk of worsening chronic conditions. It also promotes the physical and mental well-being of disabled older adults, ultimately playing a key role in alleviating anxiety symptoms in this population.

## 5. Limitations

This study had several limitations. Its cross-sectional design restricted causal inference, making it unclear whether inadequate ventilation contributed to anxiety or whether anxious individuals ventilated less. Population stratification was not fully addressed, as demographic factors such as age, gender, and socioeconomic status might have interacted with ventilation behaviors and mental health outcomes. In addition, reliance on self-reported ventilation measures introduced recall bias and lacked objective quantification of airflow. Future research was recommended to employ longitudinal or randomized designs, incorporate stratified analyses, and use objective tools such as indoor air quality sensors to capture real-time ventilation duration, window-opening frequency, and airflow metrics, thereby improving measurement accuracy and causal interpretation.

## 6. Conclusion

This study provided empirical evidence that frequent indoor window ventilation was associated with reduced anxiety symptoms among older adults with disabilities in China. Using nationally representative data, the findings demonstrated that ventilation served as a simple, low-cost, and environmentally sustainable strategy that promoted mental health in this vulnerable population. Consistent protective effects were observed across different ventilation frequencies and seasonal patterns, underscoring the importance of incorporating indoor air practices into public health initiatives. These results highlighted the need for policy attention and community-level interventions, including awareness campaigns and improvements to residential environments, to support healthier aging.

## Acknowledgments

We thank all of the Peking University for making the CLHLS data publicly available.

## Author contributions

**Conceptualization:** Qiuhua Zhang, Zishuo Huang, Tingke Xu.

**Supervision:** Qiuhua Zhang, Zishuo Huang, Tingke Xu.

**Visualization:** Qiuhua Zhang, Qi Zheng, Zishuo Huang, Tingke Xu.

**Formal analysis:** Zishuo Huang.

**Methodology:** Zishuo Huang, Tingke Xu.

**Writing – original draft:** Qiuhua Zhang, Qi Zheng, Zishuo Huang, Tingke Xu.

**Writing – review & editing:** Qiuhua Zhang, Qi Zheng, Zishuo Huang, Tingke Xu.
